# ¿Do immigrant psychotic patients receive less psychotherapy assessment compared to non-immigrant psychotic patients?

**DOI:** 10.1192/j.eurpsy.2021.1817

**Published:** 2021-08-13

**Authors:** A. Trabsa, L. Vargas, A. Llimona, F. Casanovas, M. Martín, A. Valiente, A. Moreno, B. Amann, V. Pérez-Solà

**Affiliations:** Inad-parc De Salut Mar, Hospital del Mar, Barcelona, Spain

**Keywords:** transcultural psychiatry, migration psychiatry, psychotherapy, psychosis

## Abstract

**Introduction:**

Migration is a highly defining life event which can lead to mental distress. It constitutes an overall risk factor for psychiatric disorders. However, psychotherapeutic treatment in immigrant patients is considered to be more complex, and the outcome appears to be less favorable than in patients without a migration background.

**Objectives:**

The aim of this study is to compare psychotherapy assessment between immigrant and non-immigrant psychotic patients in Barcelona.

**Methods:**

Patients who have presented, according DSM-V criteria, one or more non-affective psychotic episodes, were recruited in Acute and Chronic inpatients units at Hospital del Mar (Barcelona), leading to a total sample of 77 patients. Demographic characteristics of patients, clinical data and main pharmacological treatment were recorded through a questionnaire. Database information was completed with electronic medical records. Comparative analysis was performed with IBM SPSS using Chi-Square and t-Student test

**Results:**

From a total of 77 patients, 43 were immigrants and 34 were non-immigrants. From the total immigrants only 30,2% received psychotherapy compared to 79,4% from the non-immigrants. The most prevalent therapy received in both groups was cognitive behavioural therapy. From the immigrants group only 2,3% received psychoeducation compared to 11,8% from the non-immigrant group.

**Conclusions:**

According to our results, there are important and significant differences in psychotherapy assessment in migrant psychotic patients. In order to improve the mental health treatment of immigrant patients, the reasons for this poor outcome need to be investigated. These results should be considered by clinicians in order to design assessment program for this population.

**Disclosure:**

No significant relationships.

